# Associations of serum alkaline phosphatase level with all-cause and cardiovascular mortality in the general population

**DOI:** 10.3389/fendo.2023.1217369

**Published:** 2023-10-05

**Authors:** Wei Yan, Ming Yan, Hui Wang, Zilong Xu

**Affiliations:** ^1^ The First Affiliated Hospital of Harbin Medical University, Harbin, China; ^2^ The First Hospital of Harbin, Harbin, China

**Keywords:** alkaline phosphotase, NHANES, all-cause mortality, cardiovascular mortality, general population

## Abstract

**Background and aims:**

There are few population studies on the associations of serum alkaline phosphatase (AlkP) with all-cause and cardiovascular mortality. We aimed to investigate the relevancy of serum AlkP with all-cause and cardiovascular mortality in the general population.

**Methods and results:**

Our research included 34,147 adults in the National Health and Nutrition Examination Survey (NHANES) from 1999 to 2014. Cox proportional hazards regression models were used to assess the associations of serum AlkP with all-cause and cardiovascular mortality. Mediation analysis was used to analyze mechanisms that might link serum AlkP to all-cause and cardiovascular mortality. After 139.7 ± 57.8 months of follow-up, 5413 participants experienced all-cause death and 1820 participants experienced cardiovascular death. Mortality rates per 1000 person-years from various diseases increased with increasing serum concentrations of AlkP, especially all-cause death, cerebrovascular disease and cardiovascular death. High serum AlkP level significantly increased all-cause and cardiovascular mortality. After multivariate adjustment, the highest AlkP group had the highest risk to experience all-cause (hazard ratio [HR] = 1.30, *P* < 0.001) and cardiovascular mortality (HR = 1.39, *P* < 0.001) than the lowest AlkP group. γ-glutamyl transpeptidase (GGT) (13.33% and 15.79%), followed by Vitamin D (8.33% and 7.14%) and C-reactive protein (CRP) (7.69% and 10.35%) were identified as possible major mediators.

**Conclusion:**

Higher AlkP concentrations were associated with higher all-cause and cardiovascular mortality, largely related to mediated factors such as GGT, Vitamin D, and CRP. These findings suggest that lower serum AlkP level may reduce all-cause and cardiovascular mortality in general population.

## 1 Introduction

Alkaline phosphatase (AlkP) is an enzyme that catalyzes the hydrolysis of organic pyrophosphates (1). It is found in the highest concentrations in bone, liver, and kidney, and in lesser amounts in intestine, placenta, and leukocytes (1, 2). In the general population, elevation in serum AlkP level up to 1.5 times normal is considered acceptable (3). Such elevation may be physiological, as in adolescents, or pathological (4). In clinical practice, AlkP is used to aid in the diagnosis of biliary tract, liver and bone diseases (e.g., hyperparathyroidism, vitamin D deficiency, and renal osteodystrophy).

Currently, several clinical studies have reported the associations between AlkP and adverse outcomes in populations with different physio-pathological states. Among a population of older men, AlkP is positively associated with stroke events, coronary heart disease, and increased overall mortality (5). AlkP is closely associated with many adverse risk factors, especially pulmonary hypofunction, inflammation, endothelial dysfunction, and coagulation (6, 7). Among patients with chronic kidney disease (CKD), higher serum AlkP levels increase coronary calcification, cardiovascular disease (CVD) events, and overall mortality (8). Serum AlkP is also an independent predictor of mortality in patients with preserved renal function (9). Recent studies have shown that higher serum AlkP levels are associated with increased mortality in patients with diabetes mellitus and ischemic heart disease (10) and patients with hypertrophic cardiomyopathy (11). Higher serum concentrations of AlkP are also associated with greater risk of hospitalization related to fracture or infection (6). However, there are few population studies on the associations between serum AlkP and all-cause and cardiovascular mortality in the general population. The possible mechanisms are also unclear. Therefore, we examined these associations in the US population using NHANES data.

## 2 Methods

### 2.1 Study population

Data were obtained from NHANES, a program conducted by NCHS to collect information on U.S. household population health and nutrition. Details on sampling methods, survey tools and data collection are described elsewhere. All participants provided informed consent prior to participation (12).

**Figure 1:**
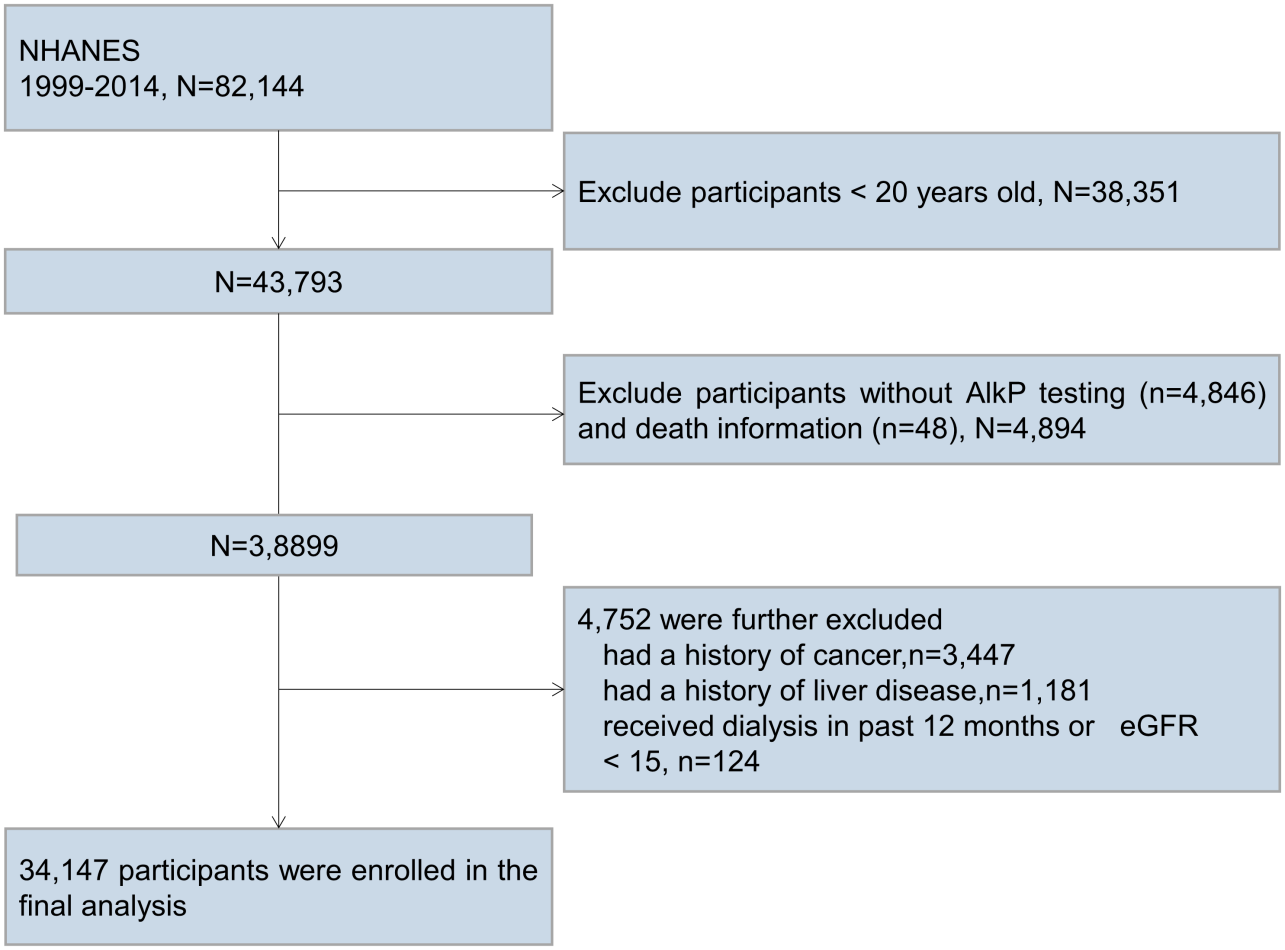
Participant flow diagram. We excluded participants who were younger than 20 years old, had no AlkP testing and death information, had a history of cancer, had a history of liver disease, and had received dialysis in the past 12 months or with eGFR < 15 mL/min/1.73m^2^.

We analyzed data for 16 consecutive years from 1999 to 2014. A total of 82,144 participants completed medical assessments at the NHANES Mobile Examination Center. Among them, participants who were younger than 20 years old (n=38351), had no AlkP testing and death information (n=4894), had a history of cancer (n=3447), had a history of liver disease (n=1181), and had received dialysis in the past 12 months or with eGFR < 15 (n=124) were excluded. Ultimately, a total of 34,147 participants were included in the current analysis (**Figure 1**).

### 2.2 Baseline data collection

Participant interviews collected self-reported data on age, race/ethnicity, gender, drinking status, smoking status, education levels, physical activity, and family poverty income ratio (PIR). Cardiovascular disease, including coronary heart disease, myocardial infarction, congestive heart failure, stroke and angina, was based on self-reported questionnaires. Diabetes was defined as a doctor-diagnosed diabetes mellitus, taking insulin, glycated hemoglobin A1c (HbA1c) level ≥ 6.5%, a fasting glucose of ≥126mg/dL (7.0mmol/L) or resting plasma glucose concentration ≥200mg/dL (11.1mmol/L). Hypertension was defined as being diagnosed by a physician and currently using of anti-hypertensive medications or resting blood pressure ≥ 140/90mmHg. Hypercholesterolemia was defined as doctor-diagnosed, currently taking cholesterol-lowering drugs, or serum total cholesterol ≥ 240mg/dL (6.2 mmol/L). The Chronic Kidney Disease Epidemiology Collaboration (CKD-EPI) equation was used to calculate glomerular filtration rate and CKD was defined as an estimated glomerular filtration rate (eGFR) of less than 60 mL/min/1.73m^2^.

Measurements of height and weight were derived from NHANES physical examination data. Blood samples were collected via venipuncture by a phlebotomist. CRP was measured using a latex-enhanced nephelometric method. Serum Albumin, alanine aminotransferase (ALT), aspartate aminotransferase (AST), γ-glutamyl transpeptidase (GGT), cholesterol, calcium, phosphorus and total bilirubin were determined with a Beckman Synchron LX20 analyzer. Albumin were measured using a bichromatic digital endpoint method. Serum ALT, AST, and GGT activities were measured using an enzymatic rate method. Serum calcium were measured using indirect (or diluted) ISE methodology. Serum phosphorus were measured using a timed-rate method. Serum total bilirubin and cholesterol was measured using a timed-endpoint method. Hemoglobin measurements were performed with a Beckman Coulter MAXM between 1999 and 2012 and the Beckman Coulter DxH 800 in 2013–2014. Serum 25(OH)D concentrations were determined by DIASORIN radioimmunoassay kit between 2001 and 2006. Starting from 2007 to 2014, they were determined by UHPLC-MS/MS method.

### 2.3 Measurement of AlkP

Serum AlkP was the exposure variable in this study. In the NHANES 1999-2001, serum AlkP activity was measured by a Hitachi Model 704 multichannel analyzer. In the NHANES 2002-2008, serum AlkP activity was measured by a Beckman Synchron LX20. In the NHANES 2009–2014, serum AlkP activity was measured by the DxC800 system. Detailed information on serum AlkP can be found in the NHANES.

### 2.4 Ascertainment of outcomes

The main outcomes of our study were all-cause and cardiovascular mortality. Mortality data were extracted based on the National Death Index (NDI) records through December 31, 2019. We divided mortality into all-causes (deaths from any cause), cerebrovascular diseases, cardiovascular diseases, nephritis, nephrotic syndrome, and nephrosis, malignancy, Alzheimer’s disease, influenza and pneumonia, chronic lower respiratory diseases, accidents (unintentional injuries), diabetes mellitus, and other causes.

### 2.5 Statistical analysis

Baseline characteristics were described according to AlkP quartiles: (1) Quartile 1 (Q1), serum AlkP concentrations ≤ 55 U/L; (2) Quartile 2 (Q2), serum AlkP concentrations between 55 and 67 U/L; (3) Quartile 3 (Q3), serum AlkP concentrations between 67 and 82 U/L; (4) Quartile 4 (Q4), serum AlkP concentration > 82 U/L. Continuous variables are expressed as mean ± standard deviation, and categorical variables are expressed as numbers with percentages. Continuous variables in baseline characteristics were analyzed by one-way ANOVA test, skewed continuous variables were analyzed by Kruskar-Wallis test, and categorical variables were analyzed by Chi-square test.

Associations of serum AlkP concentrations with mortality were assessed by Cox proportional hazards analysis. We built four models to provide statistical inference. Model 1 was only adjusted for gender, race/ethnicity and age. Model 2 was adjusted for variables in model 1 plus education level, BMI, physical activity, PIR, serum cotinine, and drinking status. Model 3 was adjusted for variables in model 2 plus CVD, diabetes, hypertension and hypercholesterolemia. Model 4 was adjusted for variables in model 3 plus eGFR, ALT, AST, total bilirubin, total calcium, phosphorus, hemoglobin, vitamin D, albumin and GGT.

The generalized linear model was applied to examine the relationships of serum AlkP levels with cardiometabolic biomarkers, including triglyceride, LDL, total cholesterol, CRP, BMI, ALT, AST, GGT, HDL, eGFR, total bilirubin, vitamin D albumin, total calcium, phosphorus and hemoglobin, adjusted for potential confounders including age, race/ethnicity, PIR, BMI, gender, education level, physical activity, serum cotinine, drinking, CVD, diabetes, hypertension, hypercholesterolemia, eGFR, ALT, AST, total bilirubin, total calcium, phosphorus, hemoglobin, vitamin D, albumin and GGT.

Restricted cubic splines (RCS) based on multivariate adjusted Cox regression visualized the linear or nonlinear associations. Based on the results of the RCS (4 knots), the associations of serum AlkP levels with mortality were examined. We drew cumulative Kaplan-Meier curves for mortality during follow-up based on predefined groups of serum AlkP concentrations. Our study used Poisson distribution to estimate the mortality rates per 1000 person-years for each cause of death across different serum AlkP levels.

A mediation analysis was conducted using a marginal structural model based on a counterfactual framework, which could decompose the total effect of a given exposure on outcomes into natural direct and indirect effects(13). The natural direct effect is the effect of AlkP on all-cause and cardiovascular mortality through pathways that does not involve related mediator variables, and the natural indirect effect represents the effect of AlkP through the pathways of mediator variables. The percentage mediated was equal to log (indirect effect)/log (total effect). For all analyses, *P* < 0.05 was considered statistically significant. All data analysis were performed by IBM SPSS statistical software (version 24.0) and R studio (version 4.1.1).

## 3 Results

### 3.1 Baseline characteristics

**Table 1** shows baseline characteristics across AlkP quartiles. The average age of the study population was 47.6 ± 17.9 years, of whom 48.2% were male. After 139.7 ± 57.8 months of follow-up, 5413 (15.9%) participants experienced all-cause death and 1820 (5.3%) participants experienced cardiovascular death. All baseline confounders were significantly different between AlkP quartiles (all *P* < 0.05). A total of 15,329 (44.9%) participants were non-Hispanic white. 9.3%, 38.3%, 14.7%, and 37.1% of participants had a history of hypertension, CVD, diabetes, and hyperlipidemia, respectively. Mean BMI was 28.8 ± 6.7 kg/m^2^. More than half (51%) of participants with AlkP serum concentrations in the lowest quartile were non-Hispanic whites. Among Mexican American participants, the proportion of the population increased with increasing serum AlkP levels. Participants with high serum AlkP levels were typically older, lower education levels, less physical activity, higher BMI, less current smokers, drank less alcohol, lower PIR, had hypertension, CVD, diabetes and hyperlipidemia, higher levels of CRP and liver function-related indicators (ALT, AST and GGT) and lower levels of VD and eGFR.

**Table 1:** Baseline characteristics of NHANES participants according to serum alkaline phosphatase.

Characteristics	Alkaline phosphatase (U/L)					P value
Number of patients	Total (n=34147)	≤55 (n=9034)	55<ALP ≤ 67 (n=8382)	67<ALP ≤ 82 (n=8339)	>82 (n=8392)	
Age, years	47.6 ± 17.9	44.3 ± 17.2	47.5 ± 17.9	48.3 ± 17.8	50.7 ± 18.2	<0.001
Male (%)	16443 (48.2)	3902 (43.2)	4277 (51.0)	4273 (51.2)	3991 (47.6)	<0.001
Race/ethnicity						<0.001
Non-Hispanic white	15329 (44.9)	4611 (51.0)	3898 (46.5)	3645 (43.7)	3175 (37.8)	
Non-Hispanic black	7072 (20.7)	1929 (21.4)	1743 (20.8)	1654 (19.8)	1746 (20.8)	
Mexican American	6596 (19.3)	1022 (11.3)	1450 (17.3)	1810 (21.7)	2314 (27.6)	
Other	5150 (15.1)	1472 (16.3)	1291 (15.4)	1230 (14.7)	1157 (13.8)	
Education levels						<0.001
Less than high school	9775 (28.7)	1764 (19.6)	2179 (26.0)	2590 (31.1)	3242 (38.7)	
High school or equivalent	7907 (23.2)	1915 (21.2)	1993 (23.8)	2000 (24.0)	1999 (23.9)	
Greater than high school	16414 (48.1)	5342 (59.2)	4197 (50.1)	3740 (44.9)	3135 (37.4)	
Physical activity						<0.001
Never	14437 (42.3)	3224 (35.7)	3401 (40.6)	3582 (43.0)	4230 (50.4)	
Moderate	9843 (28.8)	3048 (33.7)	2543 (30.3)	2275 (27.3)	1977 (23.6)	
Vigorous	9866 (28.9)	2762 (30.6)	2438 (29.1)	2481 (29.8)	2185 (26.0)	
Smoking status						<0.001
Non-smoker	8746 (25.7)	1943 (21.6)	2128 (25.5)	2317 (27.9)	2358 (28.3)	
Former smoker	18614 (54.7)	4992 (55.4)	4514 (54.0)	4498 (54.1)	4610 (55.2)	
Current smoker	6667 (19.6)	2081 (23.1)	1713 (20.5)	1495 (18.0)	1378 (16.5)	
Drinking status	22128 (70.6)	6147 (74.7)	5547 (71.9)	5405 (70.2)	5029 (65.2)	<0.001
PIR						<0.001
≤1	6677 (21.2)	1474 (17.6)	1542 (19.8)	1668 (21.8)	1993 (26.1)	
1.01-4.99	19226 (61.1)	4974 (59.3)	4824 (62.0)	4749 (62.0)	4679 (61.2)	
5	5550 (17.6)	1933 (23.1)	1412 (18.2)	1237 (16.2)	968 (12.7)	
Hypertension	3163 (9.3)	640 (7.1)	709 (8.5)	768 (9.3)	1046 (12.5)	<0.001
CVD	13090 (38.3)	2753 (30.5)	3127 (37.3)	3346 (40.1)	3864 (46.1)	<0.001
diabetic	5027 (14.7)	985 (10.9)	1109 (13.2)	1250 (15.0)	1683 (20.1)	<0.001
Hypercholesterolemia	12677 (37.1)	2843 (31.5)	3072 (36.7)	3218 (38.6)	3544 (42.3)	<0.001
BMI, kg/m^2^	28.8 ± 6.7	27.3 ± 6.2	28.7 ± 6.6	29.2 ± 6.6	29.9 ± 7.0	<0.001
Triglyceride, mmol/L	1.3 (0.9-1.9)	1.1 (0.8-1.6)	1.2 (0.9-1.8)	1.3 (1.0-2.0)	1.4 (1.0-2.0)	<0.001
LDL, mmol/L	3.0 ± 0.9	2.9 ± 0.9	3.0 ± 0.9	3.1 ± 0.9	3.1 ± 1.0	<0.001
Total cholesterol, mmol/L	5.1 ± 1.1	4.9 ± 1.0	5.1 ± 1.1	5.2 ± 1.1	5.3 ± 1.2	<0.001
HDL, mmol/L	1.4 ± 0.4	1.5 ± 0.4	1.4 ± 0.4	1.3 ± 0.4	1.3 ± 0.4	<0.001
CRP, mg/L	2.1 (0.8-4.9)	1.3 (0.6-3.2)	1.9 (0.8-4.3)	2.4 (1.0-5.2)	3.3 (1.4-7.8)	<0.001
Vitamin D, (nmol/L)	60.0 (44.4-76.1)	63.5 (48.2-78.9)	61.0 (44.7-75.9)	60.0 (44.7-73.7)	56.5 (41.9-71.4)	<0.001
eGFR, mL/min per 1.73 m^2^	96.7 (80.4-111.9)	99.2 (82.4-113.4)	97.0 (80.8-112.6)	97.0 (81.0-112.4)	95.4 (77.6-111.5)	<0.001
Albumin, g/L	42.5 ± 3.6	42.8 ± 3.4	42.6 ± 3.4	42.6 ± 3.6	41.8 ± 4.1	<0.001
ALT, U/L	21.0 (16.0-28.0)	19.0 (15.0-26.0)	21.0 (16.0-28.0)	21.0 (17.0-28.0)	22.0 (17.0-31.0)	<0.001
AST, U/L	23.0 (19.0-27.0)	22.0 (19.0-26.0)	22.0 (19.0-27.0)	23.0 (19.0-28.0)	24.0 (20.0-28.0)	<0.001
Total calcium, mmol/L	2.4 ± 0.1	2.4 ± 0.1	2.4 ± 0.1	2.4 ± 0.1	2.4 ± 0.1	0.019
GGT, U/L	20.0 (14.0-31.0)	17.0 (12.0-25.0)	19.0 (14.0-28.0)	21.0 (15.0-32.0)	24.0 (16.0-39.0)	<0.001
Phosphorus, mmol/L	1.2 ± 0.2	1.2 ± 0.2	1.2 ± 0.2	1.2 ± 0.2	1.2 ± 0.2	<0.001
Total bilirubin, umol/L	12.0 (8.6-13.7)	12.0 (10.3-15.4)	12.0 (10.3-15.4)	12.0 (10.3-15.4)	12.0 (10.3-15.4)	<0.001
Hemoglobin, g/dL	14.2 ± 1.6	13.9 ± 1.5	14.2 ± 1.5	14.3 ± 1.6	14.2 ± 1.6	<0.001

Data are numbers (percentages). All estimates accounted for complex survey designs.

PIR, poverty-income ratio; LDL, low density lipoprotein; HDL, high density lipoprotein; CRP, C-reactive protein; BMI, body mass index; ALT, alanine aminotransferase; AST, aspartate aminotransferase; GGT, γ-glutamyl transpeptidase; CVD, cardiovascular disease; eGFR, estimated glomerular filtration rate.

### 3.2 Least squares means according to serum AlkP concentrations

Higher levels of serum AlkP were significantly correlated with higher levels of triglyceride, LDL, total cholesterol, CRP, BMI, ALT, AST and GGT, and with lower levels of HDL, eGFR, total bilirubin, vitamin D and albumin at baseline (all *P* < 0.05) (**Table 2**).

**Table 2:** Least squares means according to serum alkaline phosphatase concentrations.

Characteristics	Alkaline phosphatase (U/L)				
	≤55 (n=9034)	55<ALP ≤ 67 (n=8382)	67<ALP ≤ 82 (n=8339)	>82 (n=8392)	*P* value
Triglyceride (n=16,567), mmol/L	1.31 ± 0.02	1.51 ± 0.02	1.62 ± 0.02	1.80 ± 0.03	<0.001
LDL (n=15,865), mmol/L	2.86 ± 0.02	2.99 ± 0.02	3.07 ± 0.02	3.11 ± 0.02	<0.001
Total cholesterol (n= 34,129), mmol/L	4.94 ± 0.01	5.07 ± 0.01	5.17 ± 0.01	5.27 ± 0.01	<0.001
HDL (n=34,127), mmol/L	1.45 ± 0.00	1.37 ± 0.01	1.33 ± 0.01	1.32 ± 0.01	<0.001
CRP (n=25,222), mg/L	2.83 ± 0.12	3.86 ± 0.12	4.65 ± 0.13	6.71 ± 0.13	<0.001
BMI (n=33,576), kg/m^2^	27.45 ± 0.08	28.83 ± 0.08	29.42 ± 0.09	30.15 ± 0.09	<0.001
eGFR (n=34,146), mL/min per 1.73 m^2^	96.60 ± 0.27	95.02 ± 0.28	94.33 ± 0.29	93.15 ± 0.31	<0.001
ALT (n=34,078), U/L	22.52 ± 0.28	24.23 ± 0.29	25.78 ± 0.30	28.79 ± 0.31	<0.001
AST (n=34,073), U/L	23.87 ± 0.22	24.38 ± 0.23	25.34 ± 0.24	28.05 ± 0.25	<0.001
Total bilirubin (n=34,127), umol/L	12.71 ± 0.06	12.52 ± 0.06	12.25 ± 0.07	11.79 ± 0.07	<0.001
Total calcium (n=34,119), mmol/L	2.36 ± 0.00	2.36 ± 0.00	2.36 ± 0.00	2.36 ± 0.00	0.029
Phosphorus (n=34,140), mmol/L	1.22 ± 0.00	1.21 ± 0.00	1.21 ± 0.00	1.22 ± 0.00	0.003
Hemoglobin (n=34,093), g/dL	13.98 ± 0.02	14.24 ± 0.02	14.35 ± 0.02	14.18 ± 0.02	<0.001
Vitamin D (n=29,798), nmol/L	65.99 ± 0.29	62.85 ± 0.31	61.85 ± 0.32	58.87 ± 0.33	<0.001
Albumin (n=34,147), g/L	42.77 ± 0.04	42.49 ± 0.04	42.36 ± 0.05	41.31 ± 0.05	<0.001
GGT (n=34,146), U/L	21.18 ± 0.45	24.89 ± 0.47	28.62 ± 0.48	40.42 ± 0.51	<0.001

The least squares means (mean ± SD) was estimated using general linear model with adjustment of gender (male or female), age (continuous), race/ethnicity (non-Hispanic white, non-Hispanic black, Mexican American or other), BMI (continuous), education level (less than high school, high school or equivalent, or greater than high school), physical activity (never, moderate or vigorous), PIR (0-1.0,1.01-4.99 or 5.0), serum cotinine (> 10, LOD-10 or < LOD ng/mL), drinking status (< 12 dozen drinks/yr or ≥ 12 dozen drinks/yr), CVD (yes or no), diabetes (yes or no), hypertension (yes or no), hypercholesterolemia (yes or no), eGFR (continuous), ALT (continuous), AST (continuous), total bilirubin(continuous), total calcium (continuous), phosphorus (continuous), hemoglobin (continuous), vitamin D (continuous), albumin (continuous), GGT (continuous). This is not included in the adjusted variable when the variable itself computes least squares.

PIR, poverty-income ratio; LDL, low density lipoprotein; HDL, high density lipoprotein; CRP, C-reactive protein; BMI, body mass index; ALT, alanine aminotransferase; AST, aspartate aminotransferase; GGT, γ-glutamyl transpeptidase; CVD, cardiovascular disease; eGFR, estimated glomerular filtration rate.

### 3.3 Causes of death and mortality rates

With the exception of Alzheimer’s disease and accidents (unintentional injuries), mortality rates per 1000 person-years for other causes of death increased with increasing serum concentrations of AlkP (**Table 3**). Mortality rates were significantly elevated at high concentrations of AlkP. Especially for all-cause death, cardiovascular death and cerebrovascular disease, the highest concentration of AlkP group was more than twice the mortality rate of the lowest concentration of AlkP group. This suggests that high serum AlkP concentrations are associated with higher various cause mortality.

**Table 3:** Causes of death and mortality rates per 1,000 person-years across serum alkaline phosphatase.

	Alkaline phosphatase (U/L)			
	≤55 (n=9034)	55<ALP ≤ 67 (n=8382)	67<ALP ≤ 82 (n=8339)	>82 (n=8392)
Cause of death	Mortality rate per 1,000 person-years (95% CI)			
All-cause death	8.92 (8.36-9.52)	12.15 (11.47-12.86)	13.71 (13.00-14.47)	19.67 (18.82-20.55)
Cardiovascular death	2.34 (2.06-2.66)	3.33 (2.98-3.71)	3.76 (3.40-4.17)	5.48 (5.05-5.96)
Cerebrovascular disease	0.49 (0.37-0.65)	0.70 (0.55-0.89)	0.91 (0.74-1.12)	1.29 (1.08-1.53)
Alzheimer’s disease	0.37 (0.27-0.51)	0.50 (0.37-0.66)	0.67 (0.53-0.86)	0.57 (0.44-0.74)
Malignancy	1.93 (1.68-2.22)	2.64 (2.34-2.99)	2.66 (2.36-3.01)	3.63 (3.28-4.02)
Diabetes mellitus	0.33 (0.24-0.47)	0.40 (0.29-0.55)	0.47 (0.35-0.63)	1.02 (0.84-1.24)
nephritis, nephrotic syndrome, and nephrosis	0.17 (0.10-0.27)	0.29 (0.20-0.42)	0.30 (0.21-0.42)	0.44 (0.32-0.58)
Chronic lower respiratory diseases	0.59 (0.46-0.76)	0.62 (0.48-0.80)	0.64 (0.50-0.82)	1.18 (0.98-1.41)
Accidents (unintentional injuries)	0.34 (0.25-0.48)	0.53 (0.40-0.69)	0.41 (0.30-0.56)	0.49 (0.37-0.64)
Influenza and pneumonia	0.17 (0.10-0.27)	0.22 (0.14-0.33)	0.28 (0.19-0.40)	0.50 (0.38-0.66)
other	2.18 (1.91-2.48)	2.92 (2.60-3.28)	3.61 (3.25-4.01)	5.07 (4.65-5.53)

### 3.4 Associations between serum AlkP level and all-cause mortality

**Figure 2:**
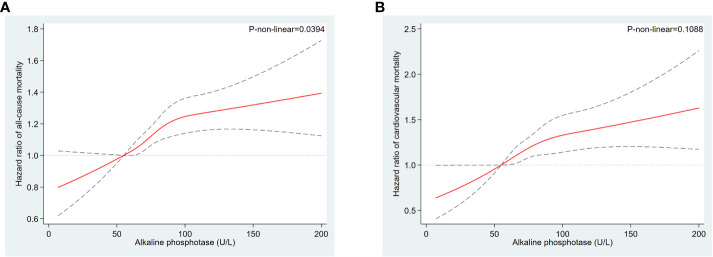
Restricted cubic spline models for the relationships between serum AlkP and all-cause and cardiovascular mortality. **(A)** all-cause mortality. **(B)** cardiovascular mortality. Hazard ratios are represented by the solid lines and the 95% CIs are represented by the dotted lines area. The model was adjusted for gender, race/ethnicity, age, education level, BMI, physical activity, serum cotinine, PIR, drinking status, CVD, diabetes, hypertension, hypercholesterolemia, eGFR, ALT, AST, total bilirubin, total calcium, phosphorus, hemoglobin, vitamin D, albumin and GGT.

**Figure 3:**
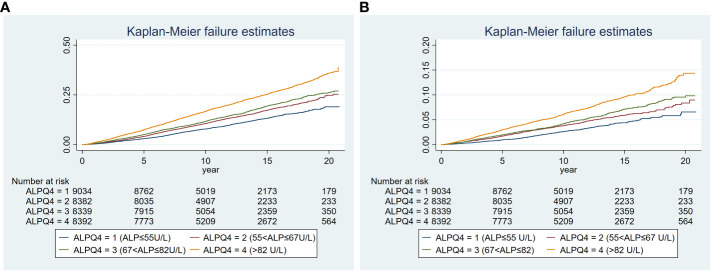
Kaplan-Meier plot of the associations of serum AlkP quartiles with all-cause and cardiovascular mortality. **(A)** all-cause mortality. **(B)** cardiovascular mortality. The model was adjusted for gender, race/ethnicity, age, education level, BMI, physical activity, PIR, serum cotinine, drinking status, CVD, diabetes, hypertension, hypercholesterolemia, eGFR, ALT, AST, total bilirubin, total calcium, phosphorus, hemoglobin, vitamin D, albumin and GGT.

All-cause mortality was higher in the higher quartiles of AlkP in all Cox regression models, with a statistically significant trend (*P* < 0.001 for trend) (**Table 4**). The higher AlkP group (Q3 and Q4) is more likely to experience all-cause mortality than the lowest group (Q1) in 4 models. In model 1, participants in Q4 group had a 57% higher risk of all-cause mortality than those in Q1 group (HR = 1.57, 95% CI: 1.45-1.70). In model 2, we further adjusted for BMI, education, physical activity, PIR, drinking status, and serum cotinine. The HR for all-cause mortality was 1.35 (95% CI: 1.23-1.48) in Q4 group compared with Q1 group. After additional adjustment for chronic diseases (including diabetes, hypercholesterolemia, hypertension, and CVD) in model 3, participants in Q4 group had a 35% higher risk of all-cause mortality (HR = 1.35, 95% CI: 1.24-1.48). After additional adjustment for eGFR, ALT, AST, total bilirubin, total calcium, phosphorus, hemoglobin, vitamin D, albumin and GGT in model 4, participants in Q4 group had a 30% higher risk of all-cause mortality than those in Q1 group (HR = 1.30, 95% CI: 1.17-1.44), and participants in Q3 group had a 23% higher risk of all-cause mortality than those in Q1 group (HR = 1.23, 95% CI: 1.11–1.36). There was no significant difference between Q2 group and Q1 group (HR = 1.07, 95% CI: 0.97–1.19). High serum AlkP concentrations remained a significant independent risk factor for all-cause mortality. The association between AlkP and all-cause mortality was non-linear (*P* < 0.05) according to RCS model (**Figure 2A**). For all-cause mortality, Kaplan-Meier analysis showed a statistically significant difference in survival probability between AlkP quartiles (**Figure 3A**). Kaplan-Meier survival curves showed that the prognosis of the high serum AlkP concentration group was generally worse than that of the low serum AlkP concentration group.

**Table 4:** Cox regression models for the association between serum alkaline phosphatase groups and clinical outcomes accounting for complex survey design.

	Q1	Q2		Q3		Q4	P value	P trend
		HR (95%CI)	P value	HR (95%CI)	P value	HR (95%CI)		
All-cause mortality								
unadjust (n=34,147)	Ref	1.35(1.24-1.47)	<0.001	1.51(1.39-1.65)	<0.001	2.15(1.98-2.32)	<0.001	<0.001
model l (n=34,147)	Ref	1.09(1.00-1.19)	0.052	1.27(1.17-1.38)	<0.001	1.57(1.45-1.70)	<0.001	<0.001
model 2 (n=28,525)	Ref	1.04(0.95-1.15)	0.415	1.17(1.07-1.29)	0.001	1.35(1.23-1.48)	<0.001	<0.001
model 3 (n=28,410)	Ref	1.05(0.95-1.16)	0.319	1.19(1.08-1.31)	<0.001	1.35(1.24-1.48)	<0.001	<0.001
model 4 (n=24,865)	Ref	1.07(0.97-1.19)	0.178	1.23(1.11-1.36)	<0.001	1.30(1.17-1.44)	<0.001	<0.001
CVD mortality								
unadjust (n=34,147)	Ref	1.41(1.21-1.64)	<0.001	1.63(1.40-1.88)	<0.001	2.33(2.03-2.68)	<0.001	<0.001
model l (n=34,147)	Ref	1.11(0.96-1.30)	0.162	1.35(1.16-1.57)	<0.001	1.65(1.44-1.90)	<0.001	<0.001
model 2 (n=28,525)	Ref	1.01(0.85-1.20)	0.893	1.23(1.04-1.45)	0.013	1.42(1.21-1.65)	<0.001	<0.001
model 3 (n=28,410)	Ref	1.02(0.86-1.21)	0.854	1.25(1.06-1.47)	0.008	1.41(1.21-1.65)	<0.001	<0.001
model 4 (n=24,865)	Ref	1.06(0.88-1.27)	0.548	1.33(1.11-1.59)	0.002	1.39(1.16-1.66)	<0.001	<0.001

Model 1 is adjusted for gender (male or female), age (continuous) and race/ethnicity (non-Hispanic white, non-Hispanic black, Mexican American or other).

Model 2 is adjusted for variables in model 1+ BMI (continuous), education level (less than high school, high school or equivalent, or greater than high school), physical activity (never, moderate or vigorous), PIR (0-1.0,1.01-4.99 or 5.0), serum cotinine (> 10, LOD-10 or < LOD ng/mL) and drinking status (< 12 dozen drinks/yr or ≥ 12 dozen drinks/yr).

Model 3 is adjusted for variables in model 2 + CVD (yes or no), diabetes (yes or no), hypertension (yes or no) and hypercholesterolemia (yes or no).

Model 4 is adjusted for variables in model 3 + eGFR (continuous), ALT (continuous), AST (continuous), total bilirubin(continuous) total calcium (continuous) phosphorus (continuous), hemoglobin (continuous), vitamin D (continuous), albumin (continuous) and GGT (continuous).

PIR, poverty-income ratio; BMI, body mass index; ALT, alanine aminotransferase; AST, aspartate aminotransferase; GGT, γ-glutamyl transpeptidase; CVD, cardiovascular disease; eGFR, estimated glomerular filtration rate.

### 3.5 Associations between serum AlkP level and cardiovascular mortality

Cardiovascular mortality was higher in the higher quartiles of AlkP in all Cox regression models, with a statistically significant trend (*P* < 0.001 for trend) (**Table 4**). The higher AlkP group (Q3 and Q4) is more likely to experience cardiovascular mortality than the lowest group (Q1) in 4 models. In model 1, participants in Q4 group had a 65% higher risk of cardiovascular mortality than those in Q1 group (HR = 1.65, 95% CI: 1.44-1.90). In model 2, we further adjusted for BMI, education, physical activity, PIR, drinking status, and serum cotinine. The HR for cardiovascular mortality was 1.42 (95% CI: 1.21-1.65) in Q4 group compared with Q1 group. After additional adjustment for chronic diseases (including diabetes, hypercholesterolemia, hypertension, and CVD) in model 3, participants in Q4 group had a 41% higher risk of cardiovascular mortality (HR = 1.41, 95% CI: 1.21-1.65). After additional adjustment for eGFR, ALT, AST, total bilirubin, total calcium, phosphorus, hemoglobin, vitamin D, albumin and GGT in model 4, participants in Q4 group had a 39% higher risk of cardiovascular mortality than those in Q1 group (HR = 1.39, 95% CI: 1.16-1.66), and participants in Q3 group had a 33% higher risk of cardiovascular mortality than those in Q1 group (HR = 1.33, 95% CI: 1.11–1.59). There was no significant difference between Q2 group and Q1 group (HR = 1.06, 95% CI: 0.88–1.27). High serum AlkP concentrations remained a significant independent risk factor for cardiovascular mortality. The association between AlkP and cardiovascular mortality was non-linear (p = 0.1088) according to RCS model (**Figure 2B**). For cardiovascular mortality, Kaplan-Meier analysis showed a statistically significant difference in survival probability between AlkP quartiles (**Figure 3B**). Kaplan-Meier survival curves showed that the prognosis of the high serum AlkP concentration group was generally worse than that of the low serum AlkP concentration group.

### 3.6 Mediation analysis

When analyzing biological mechanisms that might link serum AlkP to mortality, we found that GGT contributed to the largest mediated effect (**Table 5**), accounting for 13.33% and 15.79% of all-cause and cardiovascular mortality, respectively. This was followed by vitamin D, which mediated 8.33% and 7.14%, respectively. Then CRP mediated 7.69% and 10.35%, respectively. Total cholesterol mediated 7.14% and 3.03%, respectively. LDL mediated 6.25% and 3.45%, respectively. Albumin mediated 5.09% and 5.56%, respectively. Total calcium, phosphorus and hemoglobin explained 5.72%, 2.94%, and 3.03% of the relationships between serum AlkP and cardiovascular mortality, respectively. GGT followed by vitamin D and CRP were identified as possible major mediators between serum AlkP and mortality.

**Table 5:** Mediation analysis^a^.

	Total effect				Indirect effect				Direct effect				%mediated^b^
	OR	Lower	Upper	P-value	OR	Lower	Upper	P-value	OR	Lower	Upper	P-value	
		95% CI	95% CI			95% CI	95% CI			95% CI	95% CI		
All-cause mortality													
Triglyceride, mmol/L	1.00016	1.00006	1.00022	<0.001	1.00000	1.00000	1.00001	0.280	1.00016	1.00006	1.00022	<0.001	0.00%
LDL, mmol/L	1.00016	1.00007	1.00022	0.006	1.00001	1.00000	1.00002	0.098	1.00015	1.00006	1.00022	0.010	6.25%
Total cholesterol, mmol/L	1.00014	1.00007	1.00019	<0.001	1.00001	1.00000	1.00001	0.002	1.00013	1.00006	1.00019	<0.001	7.14%
HDL, mmol/L	1.00013	1.00006	1.00019	0.002	1.00000	0.99999	1.00000	0.210	1.00014	1.00007	1.00019	0.002	0.00%
CRP, mg/L	1.00013	1.00003	1.00021	0.018	1.00001	1.00000	1.00002	0.024	1.00012	1.00002	1.00021	0.034	7.69%
BMI, kg/m^2^	1.00014	1.00007	1.00019	0.002	1.00000	1.00000	1.00000	0.984	1.00014	1.00007	1.00019	0.002	0.00%
eGFR, mL/min per 1.73 m^2^	1.00013	1.00006	1.00019	0.002	1.00000	1.00000	0.00000	0.108	1.00013	1.00006	1.00019	0.002	0.00%
ALT, U/L	1.00013	1.00007	1.00019	<0.001	1.00000	0.99999	1.00000	0.136	1.00014	1.00007	1.00019	<0.001	0.00%
AST, U/L	1.00014	1.00007	1.00019	<0.001	1.00000	1.00000	0.00000	0.558	1.00014	1.00007	1.00019	<0.001	0.00%
Total bilirubin, umol/L	1.00013	1.00006	1.00019	<0.001	1.00000	0.99999	1.00000	0.010	1.00014	1.00006	1.00019	<0.001	0.00%
Total calcium, mmol/L	1.00014	1.00006	1.00019	0.002	1.00000	1.00000	1.00001	0.270	1.00014	1.00006	1.00019	0.002	0.00%
Phosphorus, mmol/L	1.00014	1.00007	1.00019	<0.001	1.00000	1.00000	1.00001	0.136	1.00014	1.00006	1.00019	<0.001	0.00%
Hemoglobin, g/dL	1.00014	1.00007	1.00019	<0.001	1.00000	1.00000	1.00001	0.252	1.00013	1.00007	1.00019	<0.001	0.00%
Vitamin D, nmol/L	1.00036	1.00024	1.00047	<0.001	1.00003	1.00002	1.00005	0.000	1.00032	1.00020	1.00044	<0.001	8.33%
Albumin, g/L	1.00059	1.00050	1.00066	<0.001	1.00003	1.00001	1.00005	0.002	1.00055	1.00047	1.00063	<0.001	5.09%
GGT, U/L	1.00015	1.00009	1.00020	<0.001	1.00002	1.00000	1.00004	0.030	1.00013	1.00006	1.00018	<0.001	13.33%
Cardiovascular diseases													
Triglyceride, mmol/L	1.00016	1.00007	1.00022	<0.001	1.00000	1.00000	1.00001	0.394	1.00016	1.00007	1.00022	0.002	0.00%
LDL, mmol/L	1.00029	1.00011	1.00046	0.004	1.00001	0.99999	1.00002	0.366	1.00029	1.00010	1.00046	0.004	3.45%
Total cholesterol, mmol/L	1.00033	1.00021	1.00045	<0.001	1.00001	1.00000	1.00002	0.008	1.00032	1.00020	1.00044	<0.001	3.03%
HDL, mmol/L	1.00033	1.00022	1.00045	<0.001	1.00000	0.99999	1.00001	0.880	1.00034	1.00022	1.00045	<0.001	0.00%
CRP, mg/L	1.00029	1.00011	1.00045	0.002	1.00003	1.00001	1.00006	0.002	1.00026	1.00007	1.00042	0.006	10.35%
BMI, kg/m^2^	1.00032	1.00019	1.00043	<0.001	0.99999	0.99998	1.00000	<0.001	1.00033	1.00021	1.00044	<0.001	-3.13%
eGFR, mL/min per 1.73 m^2^	1.00033	1.00021	1.00044	<0.001	1.00000	0.99999	1.00000	0.104	1.00033	1.00021	1.00044	<0.001	0.00%
ALT, U/L	1.00032	1.00020	1.00044	<0.001	1.00000	0.99999	1.00000	0.340	1.00033	1.00020	1.00045	<0.001	0.00%
AST, vU/L	1.00033	1.00020	1.00045	<0.001	1.00000	1.00000	1.00001	0.446	1.00033	1.00020	1.00044	<0.001	0.00%
Total bilirubin, umol/L	1.00032	1.00019	1.00044	<0.001	0.99999	0.99998	1.00000	<0.001	1.00033	1.00020	1.00045	<0.001	-3.13%
Total calcium, mmol/L	1.00035	1.00023	1.00045	<0.001	1.00002	1.00001	1.00003	<0.001	1.00033	1.00021	1.00043	<0.001	5.72%
Phosphorus, mmol/L	1.00034	1.00022	1.00045	<0.001	1.00001	1.00000	1.00001	<0.001	1.00033	1.00021	1.00044	<0.001	2.94%
Hemoglobin, g/dL	1.00033	1.00021	1.00045	<0.001	1.00001	1.00000	1.00002	0.246	1.00033	1.00020	1.00044	<0.001	3.03%
Vitamin D, nmol/L	1.00014	1.00008	1.00019	<0.001	1.00001	1.00000	1.00002	<0.001	1.00013	1.00007	1.00019	<0.001	7.14%
Albumin, g/L	1.00018	1.00014	1.00022	<0.001	1.00001	0.99999	1.00002	0.426	1.00018	1.00013	1.00022	<0.001	5.56%
GGT, U/L	1.00038	1.00026	1.00047	<0.001	1.00006	1.00002	1.00010	0.010	1.00032	1.00020	1.00042	<0.001	15.79%

a Hazard ratios were adjusted for gender (male or female), age (continuous), race/ethnicity (non-Hispanic white, non-Hispanic black, Mexican American or other), BMI (continuous), education level (less than high school, high school or equivalent, or greater than high school), physical activity (never, moderate or vigorous), PIR (0-1.0,1.01-4.99 or 5.0), BMI (continuous), serum cotinine (> 10, LOD-10 or < LOD ng/mL), drinking status (< 12 dozen drinks/yr or ≥ 12 dozen drinks/yr), CVD (yes or no), diabetes (yes or no), hypertension (yes or no), hypercholesterolemia (yes or no), eGFR (continuous), ALT (continuous), AST (continuous), total bilirubin(continuous), total calcium (continuous), phosphorus (continuous), hemoglobin (continuous), vitamin D (continuous), albumin (continuous), GGT (continuous). This is not included in the adjusted variable when the variable itself computes mediation analysis (n=24,865).

b The percentage mediated was calculated by log (indirect effect)/log (total effect).

PIR, poverty-income ratio; LDL, low density lipoprotein; HDL, high density lipoprotein; CRP, C-reactive protein; BMI, body mass index; ALT, alanine aminotransferase; AST, aspartate aminotransferase; GGT, γ-glutamyl transpeptidase; CVD, cardiovascular disease; eGFR, estimated glomerular filtration rate.

## 4 Discussion

In the general U.S. adults, AlkP was associated with increased all-cause and cardiovascular mortality, largely related to factors such as GGT, vitamin D, and inflammation. The associations between higher AlkP levels and mortality were attenuated but not eliminated even after adjusting for multiple confounders including BMI, smoking status, diabetes, liver enzymes, C-reactive protein, vitamin D and so on. Higher levels of serum AlkP increase mortality per 1000 person-years for various causes of death, especially for all-cause death, cardiovascular death and cerebrovascular disease. Our results confirm some previous findings on the associations between AlkP and mortality and extend the findings to the general population. To our knowledge, this is the largest US general population study to investigate the associations between AlkP and all-cause and cardiovascular mortality.

Almost all population studies have shown that AlkP is associated with increased all-cause mortality, as observed in this study. For example, results from China National Stroke Registry in 2018 showed that among stroke patients with preserved kidney function, AlkP may be associated with stroke recurrence, poor functional outcome and all-cause mortality after stroke (9). A multicenter prospective study showed that serum AlkP level at dialysis initiation was associated with all-cause mortality during maintenance dialysis (14). Previous studies on the relation between AlkP and cardiovascular mortality are controversial. For example, in African Americans with CKD, serum AlkP was not significantly related to increased risk of cardiovascular events(15). A meta-analysis of dialysis patients showed that elevated serum AlkP significantly increased cardiovascular mortality in peritoneal dialysis patients, but did not increase cardiovascular mortality in hemodialysis patients. Elevated serum AlkP is an independent risk factor for all-cause mortality in hemodialysis or peritoneal dialysis patients. Further studies are needed to clarify the relationship between serum AlkP with cardiovascular mortality in dialysis patients (16). However, among the general US population (NHANES III) and survivors of myocardial infarction, higher levels of serum AlkP are associated with cardiovascular mortality (2). The findings of this study are consistent with our study, but we used a larger and non-specific population sample with longer follow-up, which allows the findings to be generalized to a wider population.

In addition, this study used mediation analysis to analyze potential mechanisms, and found the following mediated mechanisms: First, GGT contributed to the largest mediated effect. A recent meta-analysis found that GGT were associated with increased cardiovascular mortality (17). As a pro-oxidant with pro-inflammatory activity, GGT affects the antioxidant capacity of glutathione, which is the most important cellular antioxidant in humans. In addition, both fibrinogen and GGT have been found to contribute to atherosclerosis and plaque formation (18, 19). Second, vitamin D is another important mediating mechanism by which elevated serum AlkP is associated with mortality. Vitamin D deficiency is associated with increased mortality and elevated serum AlkP levels (20), and adjustment of serum vitamin D levels in this study also partially attenuated the association of serum AlkP with mortality (**Table 4**). Then, in our and other studies, AlkP has been shown to correlate with CRP (21). In a previous study of 1740 middle-aged individuals, elevated serum AlkP was associated with elevated serum CRP levels compared with AlkP control group (22). In vascular disease, atherosclerosis is related to an inflammatory process (manifested by elevated CRP levels), and in advanced atherosclerotic plaques, AlkP expression are increased (21). Inflammation may be another explanation for the association of elevated serum AlkP with mortality. Although these associations can be largely explained by cerebrovascular risk factors and inflammation, the relationships between AlkP and CVD events still persist after adjustment for these factors, suggesting that the associations work through different mechanisms. For example, accumulating evidence suggests that AlkP can hydrolyze pyrophosphate in blood vessel wall to stimulate vascular calcification (23, 24). In a longitudinal study of 134 patients with stage IV and V CKD, higher serum AlkP levels were associated with progressive arterial calcification (25). The mechanism is controversial and may arise from valvular heart disease and poor plaque stability (26). We were unable to confirm whether vascular calcification explained the associations, because there were no vascular calcification data in the NHANES, and this suggestion remains speculative. Other mechanisms are also possible, and future researches should focus on specific AlkP isozymes to characterize the pathophysiology linking AlkP to mortality.

The strength of this study is that it used a nationally representative sample of U.S. adults, which allows the findings to be generalized to a wider population. In order to reduce the influence of confounding factors, we adjusted for a number of potential confounding factors where possible, including multiple features known to affect cardiovascular events, as well as features associated with higher AlkP levels. In addition, this study also explored possible mechanisms for the associations of serum AlkP with mortality. However, this study also has some limitations that need to be considered. First, our findings are based on data from the US population, and the findings need to be confirmed in other populations. Second, although a large number of potential confounders have been adjusted for, some undiscovered confounders cannot be ruled out. Finally, AlkP measured in NHANES is present in serum and there are no data on AlkP isoforms, therefore, we cannot infer whether bone or hepatic origin of AlkP is associated with increased mortality.

## 5 Conclusion

In this nationally representative prospective cohort of U.S. adults, higher AlkP concentrations were associated with higher all-cause and cardiovascular mortality, largely related to mediated factors such as GGT, vitamin D, and CRP. These findings suggest that lowering serum AlkP levels may reduce all-cause and cardiovascular mortality in the general population.

## Data availability statement

The raw data supporting the conclusions of this article will be made available by the authors, without undue reservation.

## Ethics statement

The NHANES survey was approved by the National Center for Health Statistics Institutional Review Board. The study reported in this manuscript was exempt from ethical committee approval because it was based on publicly available data. NHANES has obtained the written informed consent from all participants. All procedures were performed in accordance with relevant guidelines. The studies were conducted in accordance with the local legislation and institutional requirements. The participants provided their written informed consent to participate in this study.

## Author contributions

WY designed the analysis and wrote the first draft of the article. MY conducted the model parameterization and the statistical analyses. WY, MY, HW, and ZX revised the manuscript. All authors contributed to the article and approved the submitted version.
